# *Garcinia brasiliensis* Leaves Extracts Inhibit the Development of Ascitic and Solid Ehrlich Tumors

**DOI:** 10.3390/ph18010024

**Published:** 2024-12-28

**Authors:** Lucas Sylvestre Silva, Eduardo Cavallini, Rafael André da Silva, Monielle Sant’Ana, Ariane Harumi Yoshikawa, Thiago Salomão, Bianca Huang, Paula Craice, Luiz Philipe de Souza Ferreira, Heitor Pedro Della Matta, Cristiane Damas Gil, Maria de Lourdes Gomes Pereira, Ana Paula Girol

**Affiliations:** 1 Post Graduate Program in Structural and Functional Biology, Paulista School of Medicine (UNIFESP-EPM), Federal University of São Paulo, São Paulo 04023-062, SP, Brazil; lucas.sylvestre@icloud.com (L.S.S.); educavallini01@gmail.com (E.C.); monibiologia@yahoo.com.br (M.S.); luiz.philipe@unifesp.br (L.P.d.S.F.); cristiane.gil@unifesp.br (C.D.G.); 2 Department of Biology, Institute of Biosciences, Humanities and Exact Sciences (Ibilce), São Paulo State University (UNESP), São José do Rio Preto 15054-000, SP, Brazil; rafael.andre@unesp.br; 3 Experimental and Clinical Research Center (CEPEC), Padre Albino University Center (UNIFIPA), Catanduva 15809-144, SP, Brazil; ariane.yoshikawa@unifipa.com.br (A.H.Y.); thi_salomao@hotmail.com (T.S.); biancafameca@gmail.com (B.H.); paulacraice@outlook.com (P.C.); heitorpedm@gmail.com (H.P.D.M.); 4 CICECO-Aveiro Institute of Materials, Department of Medical Sciences, University of Aveiro, 3810-193 Aveiro, Portugal; mlourdespereira@ua.pt

**Keywords:** *Garcinia brasiliensis*, antitumor, Ehrlich tumor, antioxidant

## Abstract

**Background:** *Garcinia brasiliensis* is traditionally known for its medicinal properties. Objectives: Here, we investigated the effects of crude extract (CE) and ethyl acetate fraction (EAF) obtained from *G. brasiliensis* leaves on the ascitic (EA) and solid (ES) forms of Ehrlich tumors. **Methods**: Induced and uninduced BALB/c mice were treated intramuscularly, for 7 or 14 days, with saline solution or CE and EAF, both at a 10% concentration, based on in vitro cytotoxicity assessment. Biochemical analyses were also performed to evaluate in vivo cytotoxicity. In relation to tumor-induced animals, morphological changes, plasma enzymes, inflammatory mediators and the induction of apoptosis were analyzed, in addition to histopathological studies, to evaluate the inhibition of tumor growth. **Results**: Alanine aminotransferase (ALT), aspartate aminotransferase (AST) and gamma glutamyl transferase (GGT) were regulated by CE and EAF administration. Furthermore, both treatments were effective in inhibiting tumor growth in EA and ES by modulating the levels of interleukin (IL)-6 and tumor necrosis factor (TNF)-α, decreasing mast cells numbers and inducing apoptosis. **Conclusions**: This research indicates that both CE and EAF from *G. brasiliensis* leaves have potential antitumor effects with low cytotoxicity.

## 1. Introduction

Cancer is a malignant disease characterized by the rapid and uncontrolled formation/division of abnormal cells that can form tumor nodules or proliferate elsewhere in the body [1]. The disease, which is considered one of the most devastating human pathologies, presents a versatile range of striking clinical features leading to millions of deaths each year with major negative implications for social, economic and health levels [2].

One of the biggest challenges in developing new drugs for cancer is the inhibition or activation of the target without interfering with normal cellular functions. Therefore, it is necessary to characterize the actions of the new therapeutic proposals and establish safe and effective dosages. Preclinical studies are indispensable, and an interesting model is the Ehrlich tumor, which originates from female mouse breast adenocarcinoma. Ehrlich tumors resemble human tumors that are more sensitive to chemotherapy, as they are undifferentiated and fast growing [3].

The experimental model of cancer using Ehrlich tumor cells is widely used to understand tumorigenic, immunological and physiological processes, and also to evaluate new diagnostic methods and therapeutic actions of drugs, since the model is similar to pathological and behavioral processes related to tumors in humans [4].

Ehrlich tumors can develop into two forms, depending on where the tumor cells are inoculated. The ascitic form develops after peritoneal inoculation where ascites is formed as a consequence of tumor-induced inflammation due to increased peritoneal vascular permeability [5]. In the solid form, in which inoculation occurs subcutaneously, the tumors are circumscribed by a defined capsule and are composed of connective tissue infiltrated by lymphocytes, polymorphonuclear and neoplastic cells. They have significant heterogeneity in cell composition and many necrotic zones, predominantly in the central areas. Projections of connective tissue from the capsule to the tumor mass are common findings [5].

Several treatments are proposed for cancer, such as chemotherapy, radiation therapy and surgery. Chemotherapy is considered an effective method for treatment; however, its success is limited due to its hepatotoxic, nephrotoxic, cardiotoxic, myelosuppressive, immunosuppressive and other cytotoxic effects [6]. Despite the advances in medical research related to cancer treatment, the development of tumor resistance against the available therapies, as well as the various side effects of chemotherapy drugs, encourages research for newer, safer and more effective drugs.

In Brazil, therapies based on medicinal plants are regulated by the National Policy on Medicinal Plants and Herbal Medicines and by the National Policy on Integrative and Complementary Practices of the Brazilian Unified Health System [7]. In view of this, there is scientific interest in investigating the effectiveness of medicinal herbs and developing herbal medicines or isolating bioactive compounds for different clinical conditions, including cancer.

In this study, we highlight a native Brazilian plant with great medicinal potential, *Garcinia brasiliensis* (Family Clusiaceae), popularly known as “Bacupari”. This arboreal and fruitful plant, with sweet, astringent and refreshing fruits, is widely distributed throughout Brazil, and is used in popular and traditional medicine to treat inflammations, infections and allergies [8,9,10].

The fruit, as well as other organs of *G. brasiliensis*, has already been the subject of studies in the last two decades, used in crude extracts, fractions, oil and/or isolated components [11]. These studies showed that G. *brasiliensis* contains important phytochemical components such as phenols and flavonoids with anti-inflammatory, anti-allergic, antioxidant, antimicrobial, antitumorigenic and photoprotective properties. The protective effects of *G. brasiliensis* were investigated in Chagas disease, schistosomiasis, leishmaniasis, wound closure, pain and inflammation modulation in obese rats [8,12,13,14,15,16,17,18].

However, its effect as an antineoplastic and pro-apoptotic potential is still little explored. The effects of the bioactive compound 7-epiclusianone isolated from *G. brasiliensis* were investigated in vitro in lung cancer cells and glioblastoma, and it was demonstrated that the compound is able to stop the cell cycle of cancer cells [19,20].

Therefore, we conducted a study on the herbal properties and cytotoxicity of extract solutions from *G. brasiliensis* leaves. We evaluated the effects of their administration on ascitic and solid Ehrlich tumors to determine whether treatment with the crude extract and ethyl acetate fraction of *G. brasiliensis* alters crucial biological processes in tumorigenesis, such as apoptosis, inflammation and cytotoxicity.

## 2. Results

### 2.1. Assessment of Total Phenolic and Flavonoid Content and Free Radical Scavenging Capacity of G. brasiliensis Leaf Extract

Phytochemical analyses were carried out in order to quantify the total content of flavonoids and phenolic compounds in the crude extract (CE) at a 10% concentration and also in the ethyl acetate fraction (EAF) at a 10% concentration.

**Figure 1:**
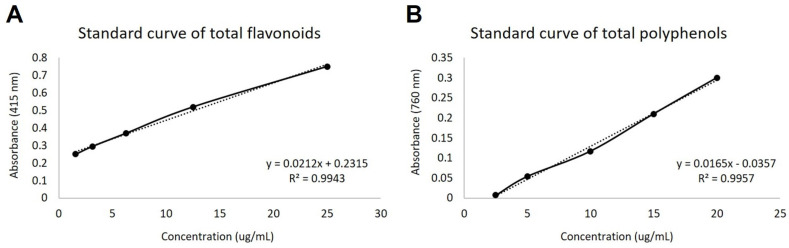
Standard curves for phytochemical analyses. (**A**) Standard curve for total flavonoid content, constructed using quercetin as standard, from a serial dilution of 25 μg/mL to 1.56 μg/mL (R^2^ = 0.9943). (**B**) Standard curve for total polyphenol content, constructed using gallic acid as standard, from different concentrations (2; 1.5; 1.0; 0.5; and 0.25 μg/mL—R^2^ = 0.9957). Solid lines represent measurements and the dashed lines represent the linear trend line.

The total flavonoid content was 94.9 μg/mL in CE and 64.24 μg/mL in EAF (Figure 1A). In turn, the polyphenol content was 10.85 μg/mL for EC and 8.42 μg/mL for EAF (Figure 1B).

**Figure 2:**
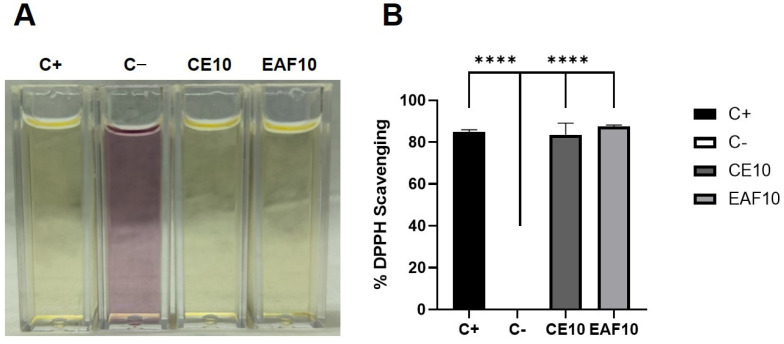
Scavenging capacity against DPPH. (**A**) DPPH assay visual result. C+: positive control (standard antioxidant, butylhydroxytoluene-BHT); C−: negative control; CE10: crude extract at a 10% concentration; and EAF10: ethyl acetate fraction at a 10% concentration. (**B**) Comparisons of total antioxidant activity among controls and CE at a 10% concentration and EAF at a 10% concentration. Statistical analysis was conducted using one-way ANOVA, followed by Tukey’s multiple comparison test. Significance levels are indicated as **** *p* < 0.0001.

In addition, through the 2,2-diphenyl-1-picryl-hydrazyl (DPPH) assay, CE at a 10% concentration and EAF at a 10% concentration showed high scavenging capacities, respectively, 83.52% ± 5.62% and 87.43% ± 0.75%, with no significant differences between them (Figure 2A,B).

### 2.2. Cytotoxicity Profile of G. brasiliensis CE and EAF

Before proceeding with the model of ascitic and solid Ehrlich tumors, it was important to determine the cytotoxicity profile of the crude extract (CE) and ethyl acetate fraction (EAF) obtained from *G. brasiliensis* leaves.

**Figure 3:**
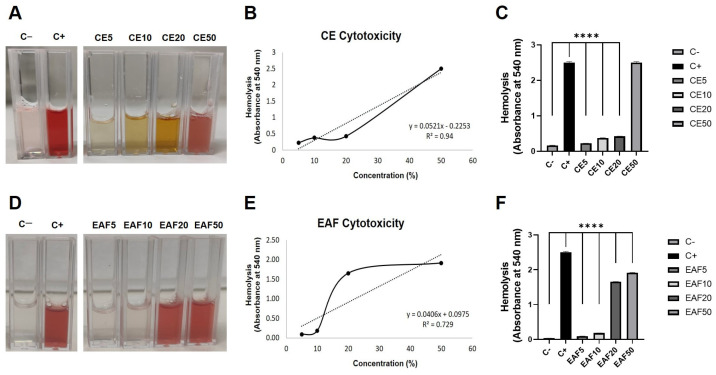
In vitro cytotoxicity test by hemolysis. (**A**) Visual result of the hemolysis test for the CE. Negative control (C−), positive control (C+), CE samples at 5% concentration (CE5), 10% concentration (CE10), 20% concentration (CE20) and 50% concentration (CE50). (**B**) Graphic of cytotoxicity in red blood cells from the CE samples. Solid line represents measurements and the dashed line represents the linear trend line. (**C**) Comparisons of cytotoxicity among controls and samples of CE. (**D**) Visual result of the hemolysis test for the EAF. Negative control (C−), positive control (C+), CE samples at 5% concentration (CE5), 10% concentration (CE10), 20% concentration (CE20) and 50% concentration (CE50). (**E**) Graphic of cytotoxicity in red blood cells from the EAF samples. Solid line represents measurements and the dashed line represents the linear trend line (**F**) Comparisons of cytotoxicity among controls and samples of EAF. Statistical analysis was conducted using one-way ANOVA, followed by Tukey’s multiple comparison test. Significance levels are indicated as **** *p* < 0.0001.

The hemolysis assay indicated that CE and EAF have low cytotoxicity at 5% and 10% concentration (*p* < 0.0001) (Figure 3A–F). The half-maximum inhibitory concentrations (IC_50_) for CE and EAF were calculated, resulting in 28.32% and 33.19%, respectively.

In this way, a concentration of 10% for CE and EAF was selected for the treatment protocols.

### 2.3. Biochemical Profile of Non-Induced and Ehrlich Tumor-Induced Animals

To evaluate the occurrence of metabolic changes caused by the administration of the extract, biochemical analyses were performed in non-induced and induced animals to ascitic and solid Ehrlich tumors. In these analyses, the treatments comprised CE at a 10% concentration and EAF at a 10% concentration and lasted 7 days for the ascitic tumor and 14 days for the solid tumor.

In this way, the plasma levels of alanine aminotransferase (ALT), aspartate aminotransferase (AST), gamma glutamyl transferase (Gamma GT), creatinine, urea, albumin, alkaline phosphatase, uric acid and blood glucose were measured.

**Figure 4:**
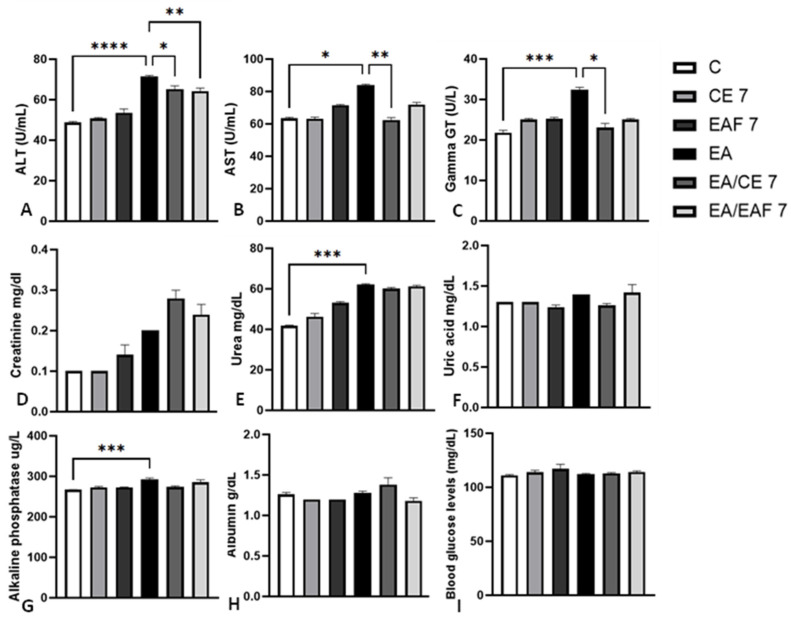
Blood biochemical analyses of ascitic Ehrlich tumor-induced animals and non-induced animals treated with CE at 10% concentration and EAF at 10% concentration for 7 days. The dosages of (**A**) ALT, (**B**) AST, (**C**) Gamma GT, (**D**) creatinine, (**E**) urea, (**F**) albumin, (**G**) alkaline phosphatase, (**H**) uric acid and (**I**) blood glucose levels were measured in blood plasma. Results presented as mean ± S.E.M (*n* = 8/group). Statistical analysis was conducted using one-way ANOVA, followed by Tukey’s multiple comparison test. Significance levels are indicated as * *p* < 0.05; ** *p* < 0.01; *** *p* < 0.001; and **** *p* < 0.0001.

**Figure 5:**
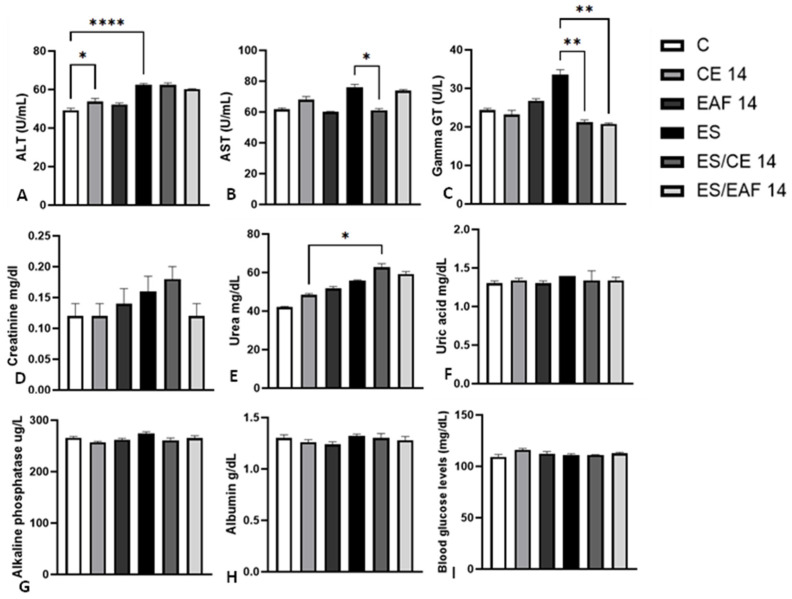
Blood biochemical analyses of solid Ehrlich tumor-induced animals and non-induced animals treated with CE and EAF for 14 days. The dosages of (**A**) ALT, (**B**) AST, (**C**) Gamma GT, (**D**) creatinine, (**E**) urea, (**F**) albumin, (**G**) alkaline phosphatase, (**H**) uric acid and (**I**) blood glucose levels were measured in blood plasma. Results presented as mean ± S.E.M (*n* = 8/group. Statistical analysis was conducted using one-way ANOVA, followed by Tukey’s multiple comparison test. Significance levels are indicated as * *p* < 0.05; ** *p* < 0.01; and **** *p* < 0.0001.

In non-induced animals but treated with CE or EAF for 7 days (CE7 and EAF7) there were no significant changes in the levels of the evaluated markers compared to group C. (Figure 4A–I). In contrast, non-induced animals and animals treated with CE for 14 days (CE14 group) showed elevated levels of ALT (*p* < 0.05) compared to those in group C (Figure 5A). However, treatment with EAF did not change the levels of markers evaluated in relation to group C (Figure 5A–I).

With regard to animals induced with ascitic Ehrlich tumors without treatment (EA group), the analyses indicated a significant increase in plasma levels of ALT (*p* < 0.0001), AST (*p* < 0.05), Gamma GT (*p* < 0.001), urea (*p* < 0.001) and alkaline phosphatase (*p* < 0.001) compared to non-induced animals (group C) (Figure 4A–C,E,G). Creatinine, uric acid, albumin and glucose dosages did not show significant changes between groups EA and C (Figure 4D,F,H,I).

Animals induced with solid Ehrlich tumors (ES group) that were not treated showed a significant increase in the plasma levels of ALT (*p* < 0.0001), when compared with animals in the control group (C) (Figure 5A), while the levels of other markers did not show significant differences between groups (Figure 5B–I).

When observing the effects of the treatments, the group induced with ascitic Ehrlich tumors and treated with CE for 7 days (EA/CE7 group) showed a reduction in ALT (*p* < 0.05), AST (*p* < 0.01) and Gamma GT (*p* < 0.05), compared to the EA group, while the other markers did not show significant changes between these groups (Figure 4A,C). On the other hand, the group induced and treated with EAF (EA/EAF7) showed a reduction only in ALT levels (*p* < 0.01), compared to the EA group (Figure 4A).

Regarding the treatment of animals induced with solid Ehrlich tumors, the group treated with CE (ES/CE14) showed a reduction in AST (*p* < 0.05) and Gamma GT (*p* < 0.01) levels, compared to the ES group (Figure 5B,C). Urea levels had a significant increase in the ES/CE14 group compared to the CE14 group (Figure 5E). On the other hand, the group induced and treated with EAF (ES/EAF14) showed a reduction in Gamma GT levels (*p* < 0.01), compared to the ES group (Figure 5C).

Finally, no differences were observed in any of the evaluated markers between the EA/CE7 and EA/EAF7 groups (Figure 4A–I) and the ES/CE14 and ES/EAF14 groups (Figure 5A–I).

### 2.4. Inhibition of the Development of Ascitic Ehrlich Tumors in Treated Animals

**Figure 6:**
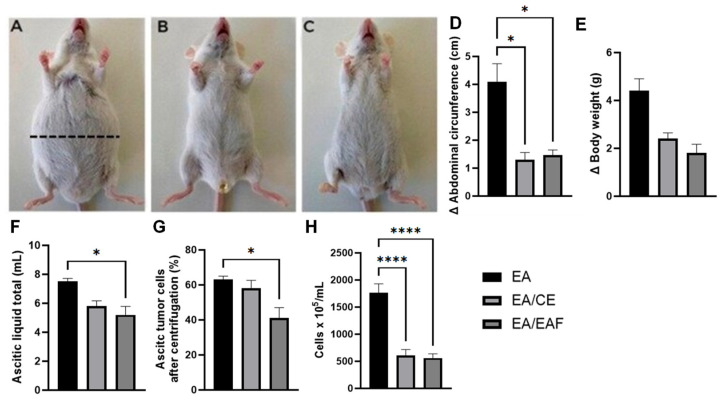
Analysis of the development of ascitic Ehrlich tumors and effects of treatments. Animals induced with ascitic Ehrlich tumors: (**A**) untreated. Dashed line represents the abdominal circumference measurement, (**B**) treated with CE for 7 days and (**C**) treated with EAF for 7 days. (**D**) Abdominal circumference (cm), (**E**) body weight (g), (**F**) ascitic liquid total (mL), (**G**) ascitic tumor cells after centrifugation (%) and (**H**) cell count × 10^5^/mL. Results presented as mean ± S.E.M. (*n* = 8/group). Statistical analysis was conducted using one-way ANOVA, followed by Tukey’s multiple comparison test. Significance levels are indicated as * *p* < 0.05; and **** *p* < 0.0001.

The animals were weighed at the beginning and at the end of the experiments to obtain their relative weights, with no significant differences between the groups (Figure 6E). However, when the tumor inhibition rate due to weight variation was calculated, the results were 45% in CE-treated animals (EA/CE group) and 59.09% in EAF-treated animals (EA/EAF group), considering moderate and high inhibition rates, respectively.

After 7 consecutive days of treatment with CE and EAF, a reduction in abdominal circumference was observed in the EA/CE (*p* < 0.05) and EA/EAF (*p* < 0.05) groups compared to the EA group (Figure 6D). The rate of tumor growth reduction in relation to abdominal circumference was 66.66% in the CE group and 67.69% in the EAF, both considered high.

Regarding the ascitic fluid, the animals in the EA/EAF group also showed a reduction in total fluid (Figure 6F) (*p* < 0.05) and ascitic cells after centrifugation (%) (Figure 6G) (*p* < 0.05) when compared to the animals in the EA group.

After counting the number of cells in the ascitic fluid, both treatments with CE (EA/CE group) and EAF (EA/EAF group) significantly decreased the number of cells (*p* < 0.0001) compared to animals of the EA group (Figure 6H).

### 2.5. Inhibition of Solid Ehrlich Tumor Development Mediated by CE and EAF Administration

**Figure 7:**
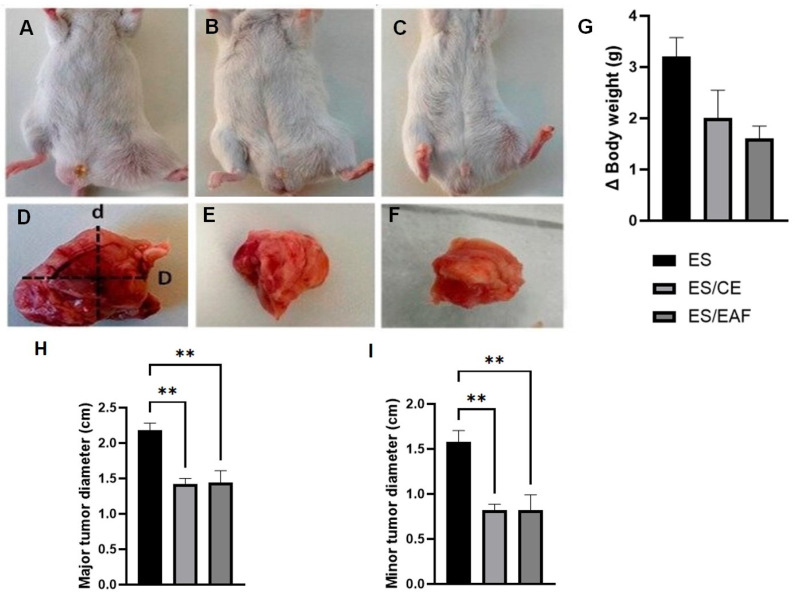
Analysis of solid Ehrlich tumor development and effects of treatments. Macroscopic analysis showing the tumors in the animals’ bodies (**A**–**C**) and after excision (**D**–**F**), with the measured axes (major D and minor d), (**G**) ∆ body weight, (**H**) major tumor diameter and (**I**) minor tumor diameter of untreated solid Ehrlich tumor-induced mice (ES) indicated in induced mice treated with crude extract (ES/CE) and induced mice treated with ethyl acetate fraction (ES/EAF). Results are presented as means ± S.E.M. (*n* = 8/group). Statistical analysis was conducted using one-way ANOVA, followed by Tukey’s multiple comparison test. Significance levels are indicated as ** *p* < 0.01.

The animals with solid Ehrlich tumors were treated for 14 consecutive days. The macroscopic analysis evidenced the reduction in the development of the tumor treated with CE and EAF in comparison to ES animals (Figure 7A–F).

The analysis of weight variation did not show a significant decrease when comparing the treated groups and the untreated animals (Figure 7G). However, in the animals induced and treated with CE, the rate of inhibition of tumor growth was 50%, while those treated with EAF had the tumor reduced by 35.7%, with percentages of inhibition being considered high and moderate, respectively.

Both treatments with CE at 10% concentration and EAF at 10% concentration reduced the major (Figure 7H) (*p* < 0.01) and minor (Figure 7I) (*p* < 0.01) tumor diameters in relation to the ES group. Likewise, the analysis of tumor showed significant differences with reduction (*p* < 0.05) in the groups treated with CE (0.7448 ± 0.04857 mm^3^) and EAF (0.7700 ± 0.1879 mm^3^) compared to the treated animals (2.684 ± 0.6847 mm^3^).

### 2.6. Histopathology Studies of Solid Ehrlich Tumors

**Figure 8:**
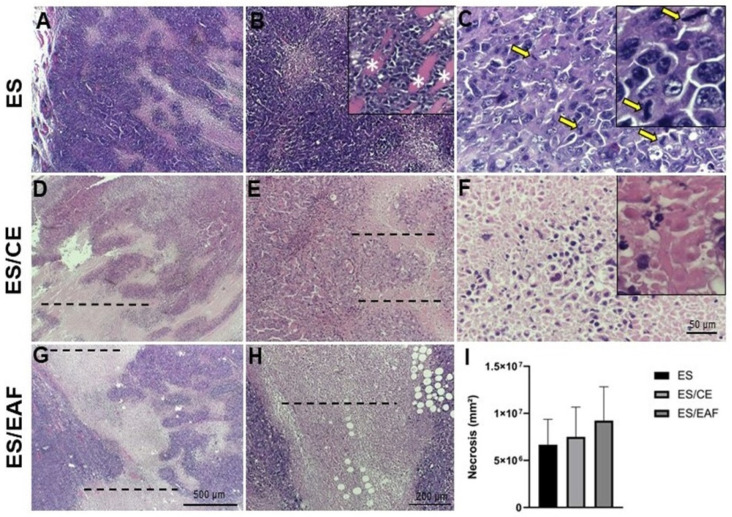
Histopathological analysis of solid Ehrlich tumors. Tumor masses of untreated animals (**A**–**C**), treated with CE (**D**–**F**) and treated with EAF (**G**–**I**). Dashed lines indicate regions of necrosis. Yellow arrows show mitosis figures. White asterisks indicate skeletal striated muscle. The detailed magnifications show mitosis figures (**C**) and necrosis regions (**F**). Bars of 500 μm (**A**,**D**,**G**), 200 μm (**B**,**E**,**H**) and 50 μm (**C**,**F**). Staining, Hematoxylin–Eosin. Statistical analysis was conducted using one-way ANOVA, followed by Tukey’s multiple comparison test.

Histopathological analysis revealed the characteristics of poorly differentiated invasive carcinoma with rapidly progressing tumor masses, frequent mitotic figures and anaplastic cells evidenced by nuclear pleomorphism, with variations in shape, size and chromatin, as well as changes in the nucleus/cytoplasm ratio, prominence and quantity of nucleoli (Figure 8C).

The invasion of the underlying skeletal muscle, leukocyte infiltration, cells in apoptosis with pycnotic nuclei and appearance of fat cells, as well as regions of necrosis presenting cells with karyolitic nuclei and acidophilus cytoplasm, were also observed (Figure 8A–C).

In the groups treated with CE (ES/CE group) and EAF (ES/EAF group), which demonstrated a macroscopic reduction in tumor masses, it was possible to observe an increase in the area of necrosis when compared to the untreated group (ES); however, the results were not statistically significant (Figure 8D–I).

### 2.7. Quantification of Mast Cells and Evaluation of Mast Cell Protease-6 (Mcpt6) Expression in Solid Ehrlich Tumors

To investigate the presence and interaction of mast cells in the development of the solid form of Ehrlich tumors, these cells were identified by histological staining with toluidine blue and quantified. Also, Mcpt6 expression was evaluated in the tumor macerate using Western blotting.

**Figure 9:**
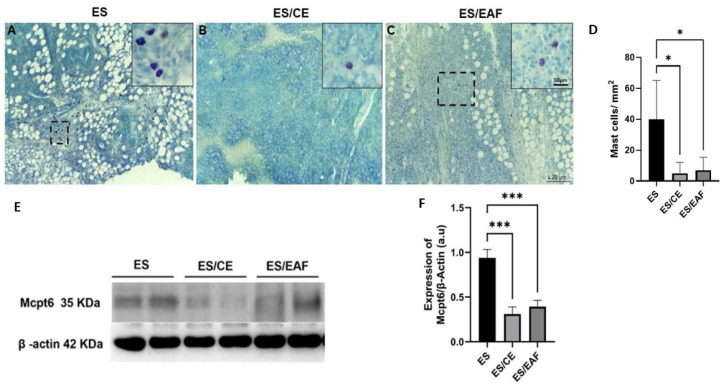
Analysis of mast cells and Mcpt6 levels in animals induced with solid Ehrlich tumor. (**A**) Various mast cells were present in animals induced with tumors without treatment. But fewer mast cells were present in animals induced with tumors and treated (**B**) with CE or (**C**) EAF. The insets represent enlargements of the dashed areas. Bars of 50 μm. Staining, Toluidine blue. (**D**) Statistical analysis was conducted using one-way ANOVA, followed by Tukey’s multiple comparison test. Significance levels are indicated as * *p* < 0.05. (**E**) Western blotting for Mcpt6 expression in animals treated with CE and EAF. (**F**) Statistical analysis was conducted using one-way ANOVA, followed by Tukey’s multiple comparison test. Significance levels are indicated as * *p* < 0.05 and *** *p* < 0.001.

Several mast cells were observed in the tumor tissues of untreated animals. In treated groups, few cells could be identified (Figure 9A–C). Animals induced with solid tumor and treated with CE and EAF (ES/CE and ES/EAF groups) showed a reduction (*p* < 0.05) of mast cells in tumor tissue, compared to animals induced but without treatment (ES group) (Figure 9D). Similarly, decreased Mcpt6 expression (*p* < 0.001) was observed in animals induced with solid tumor and treated with CE and EAF (ES/CE and ES/EAF groups) (Figure 9E,F).

### 2.8. Evaluation of Bax Expression in Ascitic and Solid Ehrlich Tumors Through Western Blotting

**Figure 10:**
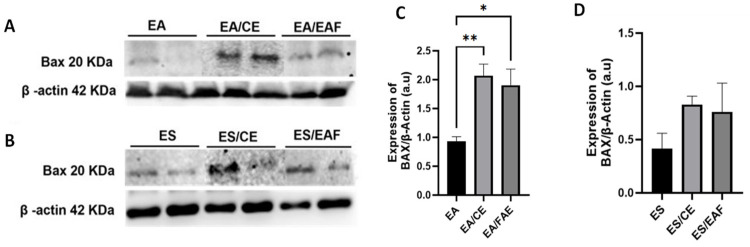
Analysis of Bax levels. Animals induced with ascitic Ehrlich tumors and treated with CE 10% concentration and EAF 10% concentration for 7 days (**A**,**C**). Animals induced with solid Ehrlich tumors and treated with CE 10% concentration and EAF 10% concentration for 14 days (**B**,**D**). Statistical analysis was conducted using one-way ANOVA, followed by Tukey’s multiple comparison test. Significance levels are indicated as * *p* < 0.05. and ** *p* < 0.01.

Higher levels of Bax were observed in animals induced with ascitic Ehrlich tumors and treated with CE at a 10% concentration (EA/CE group, *p* < 0.01) and EAF at a 10% concentration (EA/EAF group, *p* < 0.05) for 7 days, compared to untreated animals in the EA group (Figure 10A,C).

Animals induced with solid Ehrlich tumors, both the 14-day CE and 14-day EAF treatment groups, also showed an increase in Bax levels—without statistical significance, however (Figure 10B,D).

### 2.9. Measurement of Cytokine Levels IL-6, TNF-α and IFN-γ in Ascitic and Solid Ehrlich Tumors

To evaluate the influence of CE and EAF treatments on the expression of pro-inflammatory cytokines, the levels of IL-6, IFN-γ and TNF-α were measured in the ascitic fluid and tumor tissue of animals induced with ascitic and solid Ehrlich tumors.

**Figure 11:**
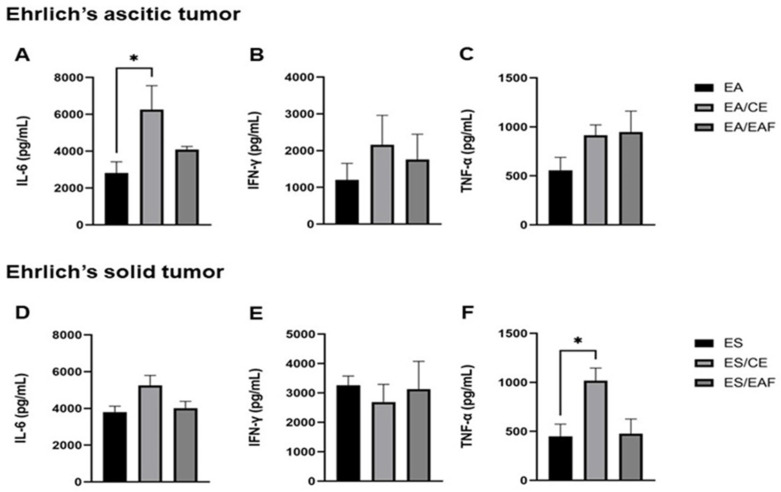
Measurement of IL-6, IFN-γ and TNF-α cytokine levels in ascitic (**A**–**C**) and solid (**D**–**F**) Ehrlich tumors. Statistical analysis was conducted using one-way ANOVA, followed by Tukey’s multiple comparison test. Significance levels are indicated as * *p* < 0.05.

In the EA/CE group, treated for 7 days, the levels of IFN-γ (Figure 11B) and TNF-α (Figure 11C) did not change significantly, while the levels of IL-6 (Figure 11A) were significantly increased (*p* < 0.05) after treatment, in comparison with the levels of such cytokines in the EA group. On the other hand, the EA/EAF group, treated for 7 days, did not present significant alterations in cytokine levels when compared with the EA group (Figure 11A–C). There was no statistically significant difference in the expression of the cytokines studied between the EA/CE and EA/EAF groups.

In the ES/CE group, treated for 14 days, the levels of IL-6 (Figure 11D) and IFN-γ (Figure 11E) did not show significant changes, while the levels of TNF-α (Figure 11F) were significantly increased (*p* < 0.05) in relation to the levels of such cytokines in the ES group. On the other hand, the ES/EAF group, treated for 14 days, did not present significant differences in cytokine levels when compared with the ES group (Figure 11D–F). Finally, there were no statistically significant differences in the expressions of the cytokines between the ES/CE and ES/EAF groups.

## 3. Discussion

**Figure 12:**
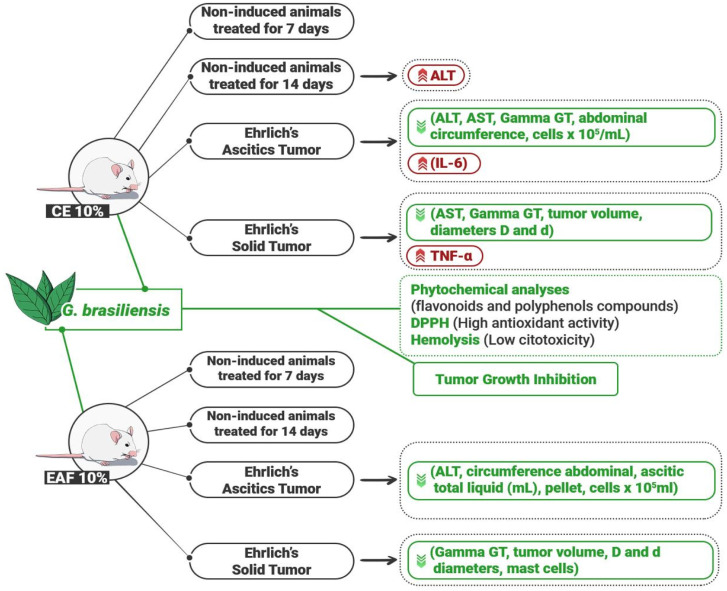
Representative scheme of the results obtained by the administration of CE at 10% concentration and EAF 10% concentration in mice non-induced and induced with ascitic and solid Ehrlich tumors. Both extractive solutions showed potential for tumor growth inhibition. Upward-facing red arrows indicate increased levels. Downward-facing green arrows indicate reduced levels.

Considering the impact of cancer on global health, as well as the importance of medicinal plants in the search for new therapies, in this investigation, we studied the effects of the administration of crude extract (CE) and ethyl acetate fraction (EAF) of *G. brasiliensis* leaves in the ascitic and solid forms of Ehrlich’s tumor. Figure 12 summarizes all the results found in this investigation.

Initially, the total content of flavonoids and polyphenols was measured, and we observed that CE and EAF at a 10% concentration exhibited high concentrations of total flavonoids and polyphenols. Furthermore, we observed that CE and EAF, both at a 10% concentration, exhibited high degrees of free radical-scavenging activity. In previous investigations, the same DPPH assay was used to assess the antioxidant potential of extracts from several plants of industrial interest [21,22]. Although it has some limitations regarding the specific conditions of the reaction, this assay can be standardized and used on a large scale, with high reproducibility between different laboratories [22,23].

We also evaluated CE and EAF through cytotoxicity analysis. The in vitro hemolysis test demonstrated that CE and EAF at a concentration of 10% had low cytotoxicity. In a previous investigation, the absence of toxicity at a concentration of 10% was also demonstrated by both hemolysis and the chorioallantoic membrane assay [18], reinforcing its safety. Therefore, a concentration of 10% for CE and EAF was selected for subsequent experiments.

Natural antioxidant components present in functional foods can protect the human body against free radical damage, which may be the reason behind several chronic diseases, including cancer [24]. The biological properties of these natural antioxidants are mostly due to their high levels of various phenolic compounds, such as flavonoids and phenolic acids [25,26].

Plants of the *Garcinia* genus are sources of important bioactive compounds, related to diverse therapeutic properties. As reviewed by Espirito Santo et al. (2020), the species *G. brasiliensis* has a high content of compounds from the classes of sesquiterpenes, biflavonoids, benzophenones, organic acids, flavonoids and xanthones, which can be obtained from leaves, fruits, fruit peels, pulp and seeds. Such compounds are especially related to anti-inflammatory, antioxidant, antiparasitic and antitumor properties [8].

The processing of plant material can be carried out using different methodologies, each with the potential to extract specific compounds. Using the high-performance liquid chromatography diode-array detection (HPLC-DAD) methodology to compare four extraction methods of bioactive compounds from the epicarp of *G. brasiliensis* fruits, it was observed that the highest levels of 7-epiclusianone and gutiferone-A were identified from the N-hexane fraction, obtained by the maceration extraction method, while higher levels of fukugetin and noratiriol were found in the ethyl acetate fraction, obtained by the extraction method with Soxhlet equipment [27].

The phenolic composition of the ethyl acetate fraction of the methanolic extract of *G. brasiliensis* leaves has also been previously studied, indicating the presence of twelve flavonoids, with emphasis on morelloflavone-7″-O-glucoside (fukugeside) and GB-2AA glucoside, which were associated with high antioxidant, antimicrobial and anti-inflammatory activity [28]. Additionally, antioxidant activity has also been characterized in the immature pulp and epicarp of *G. brasiliensis* [13,29].

In a previous investigation, our group demonstrated through phytochemical and chromatographic analyses that the CE and EAF of *G. brasiliensis* present products of plant metabolism of pharmacological interest such as polyphenols and flavonoids, comprising catechin, quercetin and garcinol as well as high antioxidant capacity [18]. Another research group demonstrated that the leaf extract of *G. brasiliensis* has a higher content of phenolic compounds and flavonoids compared to the bark and seed of *G. brasiliensis* extracts [11].

After phytochemical analyses and the selection of the CE and EAF concentration, in vivo tests were carried out. First, enzymes related to hepatic metabolism and glycemia were measured in non-induced and tumor-induced animals. Our results indicate that animals induced with ascitic Ehrlich tumors have increased plasma levels of ALT, AST, Gamma GT, alkaline phosphatase and urea. However, treatment with CE from *G. brasiliensis* promoted a reduction in ALT, AST and Gamma GT levels in these animals, indicating that the treatment can modulate the activity of important enzymes of hepatic metabolism against the development of Ehrlich’s ascitic tumor. Furthermore, treatment with EAF also promoted the reduction in ALT levels in induced animals. However, no statistically significant difference was observed in ALT levels between animals induced and treated with CE or EAF, indicating that both treatments modulate the activity of this enzyme similarly.

With regard to solid Ehrlich tumors, it was observed that the induced animals had higher levels of ALT. CE treatment promoted a reduction in AST and Gamma GT levels, but not in ALT. Furthermore, EAF treatment also reduced Gamma GT levels. Similarly to the treatment of animals with ascitic tumors, we did not observe a significant difference in the levels of the studied markers between groups of animals induced with solid tumors and treated with CE or EAF.

During the development of Ehrlich tumors, the occurrence of a metabolic imbalance is common, increasing the levels of AST, ALT, urea and creatinine [30]. One of the frequently used therapies in the treatment of tumors is radiation therapy. In the solid Ehrlich model, exposure to ionizing radiation led to increased ALT and AST levels; however, it promoted pyroptotic cell death at lower and higher doses and ferroptosis at higher doses with marked tumor reduction, which may support its use as an anti-cancer strategy [31].

Among the enzymes, ALT is more precise for liver disease than AST, as AST may also be related to muscle injuries [32], which may explain the observed effect of the treatment on the levels of such enzymes. Due to its anatomical location, the liver can be more easily affected in the development of ascitic Ehrlich tumors, which can lead to increased ALT levels. On the other hand, in the development of the solid form, the invasion of muscle tissue by the tumor occurs, which can lead to increased AST levels. Our results indicate that the proposed treatments, especially using CE, reduced ALT levels in animals induced with Ehrlich’s ascitic tumor, as well as decreased AST levels in animals induced with solid Ehrlich tumor.

The search for natural compounds that could be efficient for neoplasms face the challenge of proposing a safe treatment with less toxicity. Although some natural compounds, such as *Capsicum annuum*, show antitumor action, they can lead to metabolic imbalance, increasing AST and ALT [33]. Similarly to our findings, other researchers have verified the reduction in liver enzyme levels in the Ehrlich model after treatment with natural products such as scorpion venom [34], *Annona squamosa* extract-loaded niosome [35] and royal jelly [36].

In addition to the modulation of AST and ALT levels, we also observed that animals induced with ascitic and solid Ehrlich tumors and treated with CE showed a reduction in Gamma GT levels, as well as animals induced with solid tumors and treated with EAF, in comparison with animals induced without treatment. As reviewed by Hanigan (1998), Gamma GT levels can be increased in different types of developing tumors [37]. In a study with ascitic Ehrlich tumor models, hepatic levels of Gamma GT were reduced after treatment with alpha-lipoic acid [38]. Similarly, our results indicate that the considered treatments modulate Gamma GT levels.

After verifying the biochemical markers, we evaluated the effects of CE and EAF administration in controlling tumor growth. Our analyses indicated that treatment with CE and EAF were effective in inhibiting tumor growth in the ascitic and solid forms of Ehrlich tumors. In the ascitic model, there was a reduction in abdominal circumference and ascitic fluid volume as well as in the ascitic tumor cells numbers.

In Ehrlich tumors, the production of ascitic fluid is associated with proliferation and migration of inflammatory cells [39]. In this context, quercetin, a flavonoid with antioxidant and anti-inflammatory properties, has stood out due to its ability to inhibit the recruitment of neutrophils associated with the inflammatory process [39]. In agreement with our findings, Islam et al. (2020) also observed the inhibition of ascitic Ehrlich tumor growth, evidenced by reduced number and viability of tumor cells after treatment with leaf and bark extracts of *Morus latifolia* [40].

In the solid Ehrlich tumor model, we observed a reduction in tumor volume and major and minor tumor diameters, with a significant rate of tumor growth inhibition in the animals treated with CE and EAF. In a randomized double-blind clinical trial [41], researchers revealed that oral administration of royal jelly considerably reduced tumor size. In another study conducted by Zhang et al. (2017), results showed that royal jelly also decreased tumor weight in mice with breast tumor [42]. In addition, the use of chalcones, a bioactive compound belonging to the flavonoid family, in the treatment of Ehrlich tumors was able to reduce tumor volume [43]. Another study using mice with Ehrlich tumors, demonstrated that flavonoids, especially quercetin present in royal jelly, showed their anticancer effects through the induction of apoptosis, the reduction in animal weight and tumor volume and the inhibition of elevated serum markers such as AST and ALT [36]. In a previous study by our group, quercetin was observed in the CE and EAF extracts of leaves of *G. brasiliensis* [18].

Following our studies with solid Ehrlich tumors, we undertook the histological analyses of tumor masses, which showed anaplastic cells with invasion in skeletal muscle and necrosis regions. Although not statistically significant, the presence of large areas of necrotic tissue was observed after treatments. In addition, the increased levels of Bax in treated animals also reinforce the pro-apoptotic effect of the proposed treatments on tumor cells. The reduction in tumor masses with increased necrotic regions and apoptosis was observed in other investigations using the solid Ehrlich tumor model and treatments with extracts and bioactive compounds of medicinal plants [33,43,44]. Moreover, the modulation of apoptosis was indicated by the up-regulation of the proapoptotic genes p53, Bax [43,44] and caspase 3 as well as down-regulation of the antiapoptotic gene Bcl2 [44].

To explore the effects of treatments with CE and EAF of *G. brasiliensis* on the tumor microenvironment, the levels of pro-inflammatory cytokines IL-6, IFN-γ and TNF-α in ascitic fluid and tumor macerate were evaluated. As reviewed by Feitosa et al. (2021), the inflammatory response and the consequent expression profile of pro-inflammatory and anti-inflammatory cytokines may be variable between the two forms of Ehrlich tumors, according to the number of inoculated cells, anatomical location and other biological processes associates [5].

Our results indicated that treatment of ascitic Ehrlich tumors with CE led to increased IL-6 levels in the ascitic fluid of induced animals. Similarly, Da Cunha Leal et al. (2019) observed that the administration of high doses of gabapentin for 7 days in animals induced with ascitic Ehrlich tumors resulted in increased levels of IL-6 in the ascitic fluid compared to that in animals without treatment [45]. Another study demonstrated that natural phenolic compounds derived from bee honey, such as gallic acid and caffeic acid, induced an increase in IL-6 and IFN-γ levels in the ascitic fluid of induced animals, which contributed to the reduction in volume of the tumor and the number of viable tumor cells [46]. In line with these findings, it has already been shown that IL-6 plays an important role in the course of ascitic Ehrlich tumor.

IL-6 knockout animals induced with the disease showed greater weight gain and did not develop cachexia, compared to wild animals, indicating that cachexia induced by IL-6 secretion may be a mechanism of adaptation to cancer, leading to the increased life expectancy of animals [47]. However, some studies indicate that IL-6 levels tend to be increased after 14 days of induction of ascitic Ehrlich tumors and be reduced in the same period after treatment with *Bothrops jararaca* venom [48] and indomethacin [49]. Gentile et al. (2015) propose that IL-6 can be produced by tumor cells themselves or by cells involved in the late immune response, which justifies the increase in its secretion on day 14 after induction [49]. In the present study, we only evaluated the response to the treatment of ascitic Ehrlich tumors for 7 days; thus, the effect of prolonged treatment with CE and EAF of *G. brasiliensis* on the microenvironment of ascitic Ehlich tumor should still be investigated.

Regarding solid Ehrlich tumors, it was observed that treatment with CE induced a significant increase in TNF-α levels in the tumor. Regarding TNF-α levels, a study demonstrated that the treatment of solid Ehrlich tumors with ethanolic extract of *Salvia lachnostachys* Benth leaves also induced increased levels of this cytokine in tumor tissue, which contributed to the induction of inflammation in the tumor tissue, with consequent increase in necrosis and reduction in tumor volume and weight [50]. In contrast, using the methanolic extract of *Arthrocnemum machrostachyum* for the treatment of solid Ehrlich tumors, Sharawi (2020) observed that TNF-α levels were reduced after treatment, suggesting that such a reduction is important to prevent tumor cell survival [44]. In a review conducted by Cruceriu et al. (2020), the role of TNF-α in breast cancer was emphasized, since this cytokine can act by stimulating or inhibiting tumor progression, depending on the cellular context in which it is involved [51].

To investigate the participation of mast cells in the development of solid Ehrlich tumor, we evaluated the levels of Mcpt6; in addition, histological studies were performed with toluidine blue. In animals treated with CE and EAF, as seen through histological analysis, a reduction in the number of mast cells was observed, compared to animals that were not sujected to treatment. In addition, our results indicate that both treatments were able to reduce the levels of Mcpt6 in the tumor.

In the tumor context, mast cells can modulate several events in the tumor microenvironment, such as cell proliferation and survival, angiogenesis, invasiveness and metastasis. The increase in mast cell density may be associated with both the inhibition and stimulation of tumor progression, which demonstrates its dual role [52]. As reviewed by Ribatti et al. (2021) and Majorini et al. (2022), mast cells play an ambiguous role, depending on the type of tumor and stage of development, and is still little studied in breast tumors, with no consensus regarding their activity in these types of tumors [53,54]. Thus, mast cells have already been associated with favorable or unfavorable prognoses, in different contexts of tumor development [53,54].

Considering advances in research related to the application of natural products in diseases, including cancer, Gou et al. (2021) conducted a review regarding the relationship between mast cells, macrophages and breast cancer, proposing an investigation of the activity of natural products in inhibiting such cell types, and their relationship with breast tumor development [55]. Several studies have demonstrated the in vitro inhibition of mast cells (HMC-1 and LAD2) by various natural products. However, the authors point out that there is a lack of in vivo studies in this field [55].

Likewise, only one study indirectly investigated the participation of mast cells in solid Ehrlich tumors, through the analysis of histamine levels in the tumor microenvironment after treatment with cimetidine and/or vitamin C [56]. The researchers noted that the combined treatment of cimetidine and vitamin C, in particular, was responsible for the reduction in histamine in the tumor microenvironment, and they proposed that such a treatment, by reducing histamine levels, may reduce the activity of the PI3K/AKT/mTOR signaling pathway, which is related to the survival of breast tumor cells, with the consequent inhibition of tumor progression [56]. Similarly, our results indicate that the modulation of mast cell activity, evidenced by the reduction in these cells in the tumor tissue, is important to inhibit the growth of solid Ehrlich tumors. However, further studies are needed to better understand the role of mast cells in this type of tumor.

In summary, in this investigation, the inflammatory processes induced by the treatment, represented by the increase in IL-6 in the ascitic fluid and of TNF-α in the tumor tissue, in addition to the induction of apoptosis, represented by the increase in Bax levels, act as tumor cell inhibition mechanisms, preventing tumor progression. The inhibition of tumor growth was indicated by the reduction in abdominal circumference, total ascitic fluid volume, in addition to the number of tumor cells in animals induced with the ascitic form, as well as a reduction in volume and larger and smaller diameters of the tumor in animals induced with solid Ehrlich tumors.

Furthermore, the histopathological analysis demonstrated extensive necrosis regions in the tumor tissue of the treated animals, corroborating the proposal that the induction of inflammation, in this model, may contribute to the inhibition of tumor progression. However, we also observed a reduction in Mcpt6 levels in the microenvironment of solid Ehrlich tumors, indicating a reduction in mast cell activity in the tumors. Thus, apparently, the observed inhibitory effect from inflammation may be associated with the increased activity of other cell types that make up the immune system, which may be the target of future investigations.

## 4. Materials and Methods

### 4.1. Standardization of the Herbal Extract and Obtaining Fractions

The research is registered in the National Genetic Heritage and Associated Traditional Knowledge Management System (SisGen), Brazil, Registration AD8BA6B.

The leaves of *G. brasiliensis* were collected for exsiccates and deposited in the herbarium Irina Delanova Gemtchujnicov (BOTU nº 33511). After cleaning and drying the material, the extracts were obtained by percolation with 20 g of crushed leaves in 100 mL of 70% alcohol for 24 h [18]. The alcohol was rotaevaporated at 45 °C at reduced pressure, avoiding loss of material properties and obtaining the crude extract (CE). The extract was then diluted to a concentration of 10% (10 μg/mL) and stored in an amber vial. The solvent, ethyl acetate, was mixed with the CE at a 1:1 ratio for 24 h to obtain the organic fraction. The solvent was eliminated by evaporation and the organic portion of the fraction was re-eluted in filtered water, obtaining the ethyl acetate fraction (EAF) at a 10% concentration (7.5 μg/mL).

### 4.2. In Vitro Cytotoxicity Analysis (Hemolysis Assay)

The cytotoxicities of CE and EAF were evaluated in this investigation by the study of hemolysis. Glycosylated solution (5%) of human blood (4%) was mixed with CE and EAF at concentrations of 5%, 10%, 20% and 50%. For the positive control, 1 mL of the red blood cell suspension and 1 mL of 10% triton were added, and, for the negative control, 1 mL of the red blood cell suspension and 1 mL of glycosylated solution were added. Samples and controls were taken to the water bath at 37 °C for 15 min with subsequent centrifugation at 1500 rpm for 5 min. Finally, the samples were read in the spectrophotometer (Kasuaki IL-592-LC, Wuxi, Jiangsu, China) at 540 nm after zeroing the equipment with glycosylated solution [18]. Determinations were performed in triplicate. The IC_50_ of EC and EAF was calculated using the average of the measurements for each sample.

### 4.3. Free Radical Scavenging Capacity

DPPH was used to determine the free radical scavenging capacity of the extracts [11,18,21,22,23]. For this analysis, CE and EAF were used, both at a 10% concentration. After adding DPPH solution at 2.4 mg/100 mL, the samples were kept protected from light for 30 min for later reading in a spectrophotometer (Kasuaki IL-592-LC) at 520 nm. As a positive control, the standard antioxidant BHT was used. In the negative control, BHT, CE and EAF were omitted. Determinations were performed in triplicate. The percentage of DPPH radical elimination was estimated, according to the expression below.

$$DPPH\;Scavenging\;\left(\%\right)=\frac{negative\;control\;absorbance-sample\;absorbance}{negative\;control\;absorbance}\times 100$$

### 4.4. Analysis of Total Flavonoid and Total Polyphenol Content

To determine the total flavonoid content in CE at 10% concentration and EAF at 10% concentration, quercetin (Q4951—Sigma Aldrich, Cotia, São Paulo, Brazil) was used to generate the standard curve (serial dilution from 25 µg/mL to 1.56 µg/mL). A mixture of 1 mL of different concentrations of the curve or 1 mL of the samples added to 1 mL of 2% aluminum chloride (AlCl_3_) alcoholic solution was read at 415 nm in the spectrophotometer (Kasuaki IL-592-LC) [12,57].

The total polyphenol content in CE at 10% concentration and EAF at 10% concentration, was evaluated with Folin–Ciocalteu reagent [12,57], using galic acid (1274—Dinâmica, Indaiatuba, São Paulo, Brazil) for the construction of the standard curve (20; 15; 10; 5; 2.5 µg/mL). Sodium carbonate (NaCO_3_) (1.5 mL) and Folin–Ciocalteu reagent (0.5 mL) were added to the samples, and the absorbance was read at 760 nm using the spectrophotometer (Kasuaki IL-592-LC).

### 4.5. Ehrlich Tumors and Treatments

Male Balb/c mice aged six to eight weeks (weight between 20 and 30 g) were obtained from the Didatic and Experimental Research Unit of the Padre Albino University Center (UNIFIPA). The animals were kept in cages in an environment with controlled temperature (24° to 25 °C) with water and food ad libitum. The experimental procedures were carried out in accordance with the rules of the Ethics Committees for the Use of Animals (CEUA-UNIFESP, certificate of approval nº 7189031221, and CEUA-UNIFIPA, certificate of approval nº 15/21) and under veterinary supervision.

**Figure 13:**
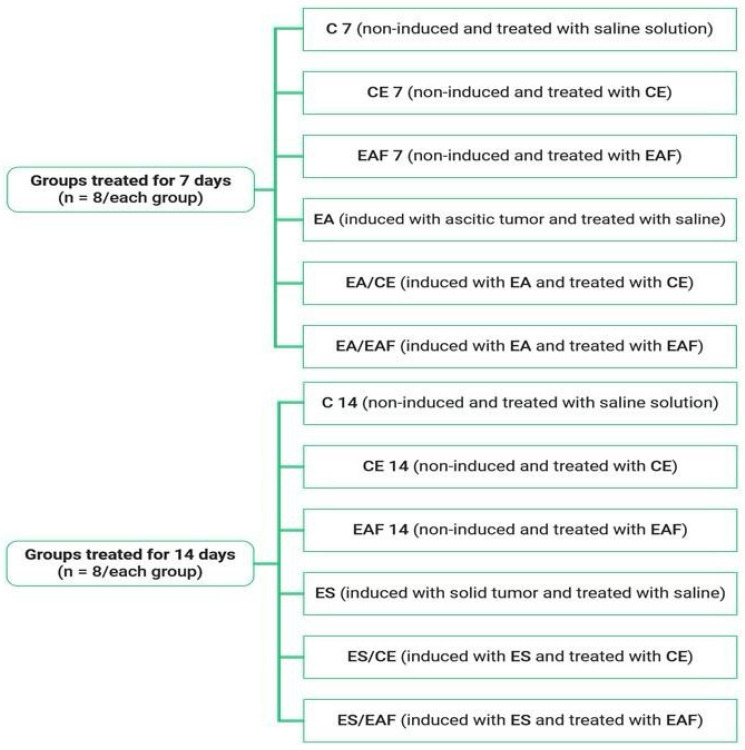
Groups of animals that were non-induced and induced with Ehrlich ascitic and solid tumors and were treated for 7 days and 14 days.

The animals were divided into 12 groups (*n* = 8/group) as follows: control animals treated with saline solution 0,9% for 7 (C 7) and 14 (C 14) days; non-induced animals treated with CE at 10% concentration and EAF at 10% concentration for 7 days (CE 7, EAF 7) and 14 days (CE 14, EAF 14); animals induced with ascitic Ehrlich tumors treated with saline solution 0.9% for 7 days (EA); animals with solid Ehrlich tumors treated with saline solution 0.9% for 14 days (ES); animals induced with ascitic Ehrlich tumors treated with CE at a 10% concentration (EA/CE) and EAF at a 10% concentration (EA/EAF) for 7 days; and animals induced with solid Ehrlich tumors treated with CE at a 10% concentration (ES/CE) and EAF at a 10% concentration (ES/EAF) for 14 days (Figure 13).

For the induction of the tumor in its ascitic form, the animals were inoculated intraperitoneally with ascitic fluid from previously induced animals. The injection was applied into the left lower quadrant and the cells amount inoculated was 0.5 mL (2 × 10^6^ cell/mL) [58]. For the solid form of the Ehrlich tumor, the inoculation of neoplastic cells was subcutaneous above the thigh in the same amount of 0.5 mL (2 × 10^6^ cell/mL) [59].

The induced animals were treated intramuscularly with 0.5 mL of CE or EAF 10% concentration, selected according to a previous study by our research group [18] and corroborated in this investigation by hemolysis assay. The treatments were started on the same day as the induction of the tumors and were continued through daily applications into the thigh region until the seventh day for the ascitic tumors [60,61] and fourteenth day for the solid tumors [62].

**Figure 14:**
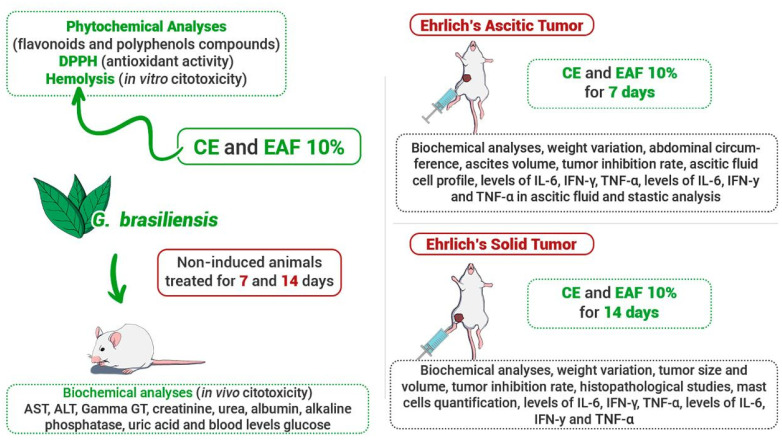
Schematic summary of the assessments used in this investigation.

The animals were euthanized by an overdose of anesthetic one day after the end of the treatment protocols. Figure 14 summarizes the analyses used in this investigation.

### 4.6. In Vivo Cytotoxicity Analysis (Biochemical Analysis of Blood)

To evaluate the in vivo cytotoxicity of CE and EAF treatments in non-induced and tumor-induced animals, the levels of alanine aminotransferase (ALT), aspartate aminotransferase (AST), gamma glutamyl transferase (Gamma GT), creatinine, urea, albumin, alkaline phosphatase, uric acid and blood glucose were measured. After euthanasia, blood was collected by cardiac puncture in heparinized syringes. After that, it was centrifuged at 7000 rpm for 15 min and the blood plasma was separated into aliquots for the analyses with commercial kits (Ortho Clinical Diagnostics, Vitros Chemistry System, San Diego, CA, USA), according to the manufacturer’s instructions.

### 4.7. Evaluation of Tumor Growth by Body Weight Variation in Ascitic and Solid Tumor

The variation in body weight was given by the difference between the final measurement (after the last day of treatment) and the initial measurement (before the start of treatment). The results are expressed in grams [62]. The percentage of inhibition of tumor growth is determined by the formula below:

$$\%\;Tumor\;Growth\;Inhibition=\frac{∆BW\;tumor\;group-∆BW\;treated\;group}{∆BW\;tumor\;group}\times 100$$

where ΔBW is the variation in body weight.

### 4.8. Evaluation of Tumor Growth by Abdominal Circumference Variation in Ascitic Tumor

The variation in the abdominal circumference measurement was calculated by the difference between the final measurement and the initial measurement, and the results were expressed in centimeters [62]. The percentage of inhibition of tumor growth is calculated according to the formula below:

$$\%\;Tumor\;Growth\;Inhibition=\frac{∆CA\;tumor\;group-∆CA\;treated\;group}{∆CA\;tumor\;group}\times 100$$

where ΔCA is the variation in abdominal circumference

For both growth evaluation techniques, the antitumor activity was classified according to the percentage of tumor growth inhibition as follows: absent = percentage of tumor inhibition is equal to 0; mild = percentage of tumor inhibition is ≤30%; moderate = percentage of tumor inhibition is ≥30–50%; high = percentage of tumor inhibition is >50% [62].

### 4.9. Evaluation of Tumor Growth by Ascites Volume

After euthanasia, ascitic fluid was aspirated from the peritoneal cavity of mice with ascitic tumors using a 5 mL syringe, and the volume was measured using a graduated centrifuge tube in mL [62].

### 4.10. Evaluation of Tumor Volume in Solid Tumors

The animals were weighed daily, and the size of the tumor measured with a caliper was determined by the following formula:

Tumor volume: 0.5 × D × d^2^ mm^3^. Here, D is the major diameter of the tumor and d the minor measured thickness [63,64].

### 4.11. Fixation, Processing and Inclusion for Light Microscopy and Histopathological Analyses

After the excision of the tumor mass, the fragments were fixed in 4% formaldehyde for 24 h and dehydrated in increasing series of ethanol, then diaphanized with xylol and included in paraffin (Lupetec, São Carlos, São Paulo, Brazil). The 3 µm sections were obtained by microtome (MRP2016SA, Lupetec, São Carlos, São Paulo, Brazil) and stained with Hematoxylin–Eosin for histopathological analyses.

The areas of necrosis were measured in different fields in the 10× objective (Leica microscope, DM500, Wetzlar, Germany), using the Leica Image Analysis software (LAS V4.0). Values are presented as mean ± SEM of the mm^2^ area obtained in the different experimental groups.

For the microscopic analysis of mast cells, 3 µm sections were stained with Toluidine blue. Mast cells were identified due to their metachromatic cytoplasmic granules. The quantification of mast cells was performed in different images per slide obtained by 10× objective and the values are expressed as number of cells per mm^2^.

### 4.12. Levels of the Cytokines IL-6, INF-γ and TNF-α by Magpix Assay

Inflammatory mediators (IL-6, INF-γ, TNF-α) were assessed using the MILLIPLEX MAP MCYTOMAG 70K kit and the LUMINEX xMAP MAGPIX instrument (Luminex, Austin, TX, USA) for the quantitative multiplex detection of multiple analytes, with speed and specificity, by means of magnetic beads. Tissue samples underwent processing, with tumor fragments macerated in liquid nitrogen for ES analysis. The ascitic fluid was centrifuged for EA analysis. Additionally, samples were treated with protease inhibitors and phosphatases. Components, including magnetic beads, controls, buffer, serum and standards, were prepared following kit instructions.

In a 96-well magnetic plate, 25 μL of standards, controls and samples were added, along with assay buffer, standards medium and magnetic beads coated with antibodies. The plate was sealed and incubated for 2 h at room temperature with agitation. Subsequent steps involved washing, incubation with detection antibodies, the addition of phycoerythrin conjugated to streptavidin and additional washing before incubation with fluid. Plate readings were performed using the LUMINEX xMAP MAGPIX, and analyte concentrations were determined using the MAGPIX xPONENT software (V4.2 NCM, Luminex, Austin, Texas, USA).

### 4.13. Western Blotting

Proteins from both EA tumor cells and ES tumor tissue were extracted using RIPA buffer (Thermo Fisher Scientific #89900, Waltham, MA, USA), supplemented with protease inhibitor (Roche #CO-RO, Basileia, Suíça) and phosphatase inhibitor (Roche #PHOSS-RO) to prevent protein degradation. The protein concentration of each sample was determined using the Qubit system (#Q33211 Quanti-iT Protein Assay Kit, Thermo Fisher Scientific). Samples containing 30 μg of total protein were separated using SDS-PAGE on a 12% gel and subsequently transferred to a nitrocellulose membrane. To prevent nonspecific binding, the membranes were blocked using a 5% solution of bovine serum albumin (BSA, Sigma-Aldrich) for a duration of 2 h. Following blocking, the membranes were incubated overnight at 4 °C with primary antibodies: goat anti-Mcpt6 Clone 286820 (R&DSystem #MAB3736, Mineápolis, MN, USA, 1:1000), rabbit anti-Bax (Abclonal #A0207, Woburn, MA, USA 1:500), and mouse anti-β-actin, clone AC-74 (Sigma-Aldrich #A2228, 1:2000). The β-actin marker was used to normalize the experiment. After incubation with primary antibodies, the membranes were washed thrice with T-TBS for 10 min each time. Subsequently, they were incubated at room temperature for 2 h with horseradish peroxidase-conjugated secondary antibodies: goat, mouse or rabbit IgG (1:3000), used for chemiluminescence detection (SuperSignal West Pico Chemiluminescent Substrate, Thermo Fisher Scientific #34578). The chemiluminescent signal was captured using UVITEC equipment (UVITEC, Cambridge, UK). For quantification, the density of the bands was analyzed utilizing ImageJ 1.53k (NIH). The ratio of the protein of interest to the housekeeping protein B-actin was calculated in order to determine relative expression levels.

### 4.14. Statistical Analysis

The results obtained were previously submitted to descriptive analysis and determination of normality. Data are presented as means ± SEM for normally distributed data and medians for non-normally distributed data. To assess normality, the Shapiro–Wilk test was performed. As the groups showed normal distribution, an ANOVA with Tukey’s post-test for multiple comparisons was employed. Data analysis was conducted using GraphPad Prism 9.0.0, utilizing either the mean or median as appropriate. Statistical significance was considered to be *p* < 0.05. Figure 14 summarizes the analyses used in this investigation.

## 5. Conclusions

Our results indicate that treatments with CE and EAF, both at a 10% concentration, were able to regulate the metabolic imbalance caused by the tumor and inhibit the growth of ascitic and solid Ehrlich tumors by inducing apoptosis and modulating mast cells and the cytokines IL-6 and TNF-α. These findings will encourage the development of new studies to deepen the understanding of the antitumor effects of *G. brasiliensis* extracts.

## Data Availability

The data used and analyzed in this study are available from the corresponding authors upon reasonable requests. The data are not publicly available due to institutional policy.
